# Peabody Developmental Motor Scales—Second Edition: A Reliable Tool for Assessing Motor Development in Children

**DOI:** 10.3390/jcm14248936

**Published:** 2025-12-18

**Authors:** Anna Chałupka-Borowska, Magdalena Sobieska

**Affiliations:** Department of Rehabilitation and Physiotherapy, Faculty of Health Sciences, Poznań University of Medical Sciences, 60-806 Poznań, Poland; msobieska@ump.edu.pl

**Keywords:** PDMS-2, motor development, assessment, reliability, validity, pediatrics

## Abstract

Early identification of motor difficulties is essential in infancy and early childhood, and current American Academy of Pediatrics recommendations emphasize that motor surveillance should accompany routine clinical visits. One standardized tool widely used for evaluating motor development is the Peabody Developmental Motor Scales–Second Edition (PDMS-2). This review summarizes the theoretical foundations and psychometric properties of the PDMS-2, the principles of administering and scoring the assessment, and evidence from validation and standardization studies conducted in different countries. A non-systematic literature search was conducted in PubMed, Scopus, and Google Scholar (2000–February 2025) using the terms “PDMS-2” OR “Peabody Developmental Motor Scales Second Edition” combined with “reliability”, “validity”, “norms”, “reference”, or “standardization”. Original and review articles published in English were included without geographical restrictions. The PDMS-2 is widely applied in both clinical and research contexts. It has been used as an outcome measure in randomized controlled trials, interventional, and observational studies involving preterm infants, children with genetic syndromes, metabolic disorders, cerebral palsy, congenital heart defects, HIV, oncological conditions, and typically developing children. Key strengths of the PDMS-2 include its broad age range, the ability to assess both gross and fine motor skills, and its quantitative scoring system, which supports diagnosis, therapeutic planning, and monitoring of developmental change. Although the tool has been validated and standardized in multiple countries, additional work is still needed to establish normative data for underrepresented populations.

## 1. Introduction

Assessment of motor development in early childhood constitutes a key component of functional diagnostics, prediction of developmental outcomes, and planning effective therapeutic interventions. The need for systematic evaluation of children’s motor competencies has been emphasized by numerous international organizations, including the American Academy of Pediatrics (AAP) and the World Health Organization (WHO). Clinical guidelines recommend assessing children at 9, 18, and 30 months of age, as well as between 4 and 5 years, just before entering school education [1]. In both clinical and research practice, several standardized tools are used, such as the Alberta Infant Motor Scale (AIMS), the Bayley Scales of Infant and Toddler Development (BSID), and the Peabody Developmental Motor Scales—Second Edition (PDMS-2). PDMS-2 is one of the most widely used tests for assessing motor development in children from birth through 5 years of age [2,3,4,5].

The aim of this article is to present the work related to the standardization of the PDMS-2, its cultural adaptations across countries, and to discuss its properties, applications in clinical practice, and its use in research—particularly in assessing children at risk for neurodevelopmental disorders.

## 2. Materials and Methods

This article is not a systematic review; however, the literature search strategy is described below. A database search was conducted in PubMed, Scopus, and Google Scholar in February 2025 using the keyword combinations: “PDMS-2” or “Peabody Developmental Motor Scales Second Edition” and “reliability” or “validity”, and “PDMS-2” and “norms” or “reference” or “standardization”. Original and review articles published in English between 2000 and 2025 were included. Additionally, references cited in identified articles were screened. Studies in which PDMS-2 was used solely for validating other tools were excluded. This article also includes a review of literature describing the use of the PDMS-2 as an outcome measure in various clinical populations. For this part of the review, systematic reviews, original studies, randomized controlled trials (RCTs), and observational studies conducted over the last 20 years were included.

## 3. Peabody Developmental Motor Scales—Second Edition (PDMS-2)

Contemporary approaches to assessing motor development in children draw upon two key theoretical frameworks. Historically, the neuromaturational model, developed by Gesell, McGraw and Shirley, was dominant. This model posits that motor development results primarily from the maturation of the central nervous system (CNS) and assumes a relatively fixed and predictable sequence of milestone acquisition, with stable transitions between successive developmental stages—from simple to more complex movements, in a cephalocaudal and proximodistal direction. In contrast, Esther Thelen and colleagues proposed the Dynamic Systems Theory (DST), which conceptualizes motor development as emerging from the continuous interaction of multiple subsystems: biological (e.g., CNS maturation, muscle strength, postural control), environmental (e.g., opportunities for exploration, availability of sensory input), and task-related. From the DST perspective, variability, non-linearity, and individually differentiated developmental trajectories are normative features of the developmental process rather than deviations. Longitudinal studies in infancy support this view, demonstrating substantial intra-individual variability in motor performance over time, even among typically developing children [6,7,8,9,10].

Both theoretical models provide an important conceptual foundation for the Peabody Developmental Motor Scales—Second Edition (PDMS-2). Although the scale originates from the neuromaturational tradition, its structure partly reflects contemporary understanding of developmental complexity: the tasks in PDMS-2 are not arranged in a strict hierarchical sequence representing a universal developmental order, but instead constitute sets of skills that allow for the assessment of a child’s current level of functioning across different motor domains. This approach is consistent with DST principles, which emphasize that development may progress in multiple directions and that various skills may emerge at different rates depending on the interaction of biological and environmental influences [6].

PDMS-2 is a standardized instrument designed to assess motor development in children from birth to 71 months of age. The scale was developed by Michael R. Folio and Rebecca R. Fewell as a continuation of earlier versions of the Peabody scales, and its second edition was published in 2000 [5,6].

PDMS-2 was created as a practical tool intended for use by therapists and specialists working with young children, with the goal of providing objective and repeatable assessment of motor skills. The manual includes detailed scoring criteria, descriptions of starting positions, and the materials required for each item, contributing to high test–retest reliability and scoring consistency. Assessment focuses on movement quality, precision, and the degree to which the motor goal is achieved [5,6,11].

The PDMS-2 was developed to (1) estimate a child’s motor competence; (2) compare gross and fine motor performance; (3) provide qualitative and quantitative information about individual motor skills; (4) monitor a child’s progress; and (5) serve as a research tool [4]. The scale enables tracking motor progress over time—both qualitatively and quantitatively. Its normative nature allows clinicians to determine whether a child’s development is within the expected range for their age. A score below two standard deviations may indicate a developmental delay requiring further diagnostic assessment or the initiation of therapeutic intervention. Importantly, the PDMS-2 allows the identification of both strengths and areas requiring support, making it valuable for designing individualized therapy plans. It may also be used for evaluating the effectiveness of interventions and comparing outcomes in clinical and research settings. Owing to its high sensitivity and precision, PDMS-2 is widely used both clinically and scientifically [6].

The Third Edition of the Peabody Developmental Motor Scales (PDMS-3) was released in 2023, introducing updated norms and a revised subtest structure. However, empirical research applying the PDMS-3 is still limited, and most clinical and psychometric evidence available in the literature concerns the PDMS-2; therefore, this review focuses on the second edition [12].

## 4. Assessment of Motor Development Using the PDMS-2

The PDMS-2 assesses both gross and fine motor skills, providing a comprehensive evaluation of a child’s motor abilities. The scale consists of six subtests: Reflexes—assesses primitive reflexes in children up to 12 months of age (8 items), Stationary—evaluates postural control and balance (30 items), Locomotion—assesses movement skills such as crawling, walking, running, and jumping (89 items), Object Manipulation—evaluates throwing, catching, and kicking (from 12 months; 24 items), Grasping—assesses development of hand grasp and fine hand movements (26 items), Visual–Motor Integration—evaluates hand–eye coordination and integration of visual and motor skills (72 items) [5,6,11].

Practical use of the PDMS-2 requires preparing specific test materials (e.g., balls, steps, small objects, blocks) and providing an environment that allows comfortable observation. Assessment is conducted individually, in a child-friendly setting, with flexibility in task order depending on the child’s functioning and mood. Testing typically takes 30–60 min. The examiner observes spontaneous responses to presented tasks and may repeat instructions or demonstrate movement patterns as allowed by the manual [6].

Testing begins with identifying the appropriate entry point (basal level) for each subtest based on the child’s chronological age or estimated ability level. The basal level ensures that testing starts at a level considered appropriate for the child’s skills. Each item is scored using a three-point scale: 0 points—does not attempt or cannot perform the task, 1 point—partial performance, 2 points—full and correct performance according to instructions. Testing continues until the ceiling level is reached—defined as the point at which the child can no longer successfully perform subsequent tasks. Raw scores are calculated for each subtest and then converted to standard scores using normative tables [6].

Based on the subtest scores, three composite indices are calculated: Gross Motor Quotient (GMQ), Fine Motor Quotient (FMQ), and Total Motor Quotient (TMQ), providing an overall picture of the child’s motor development. Raw scores are standardized and converted into percentiles, enabling comparison with population norms [5,6,11].

In clinical practice, the PDMS-2 is widely used by pediatric physiotherapists and occupational therapists to identify motor delays, determine functional strengths and weaknesses, and guide individualized intervention planning. The tool is practical for routine clinical assessment because it provides standardized scores as well as qualitative observations that help clinicians set treatment goals and track changes over time. Although no modified clinical version of the PDMS-2 exists, the original scale is frequently applied in everyday practice within its normative age range (0–71 months). Its structured scoring system and clear item descriptors support decision-making and communication with parents and multidisciplinary teams.

Although the present review focuses on the PDMS-2, it is important to acknowledge that other motor assessment tools such as the AIMS, BSID-III, and TGMD are also widely used in pediatric practice. These instruments differ in scope and purpose: for example, the AIMS focuses exclusively on infants and evaluates primarily gross motor milestones, whereas the BSID-III assesses motor skills within a broader developmental framework. A detailed comparison of these tools lies beyond the scope of this review; however, recognizing their complementary roles provides useful context for interpreting the strengths of the PDMS-2 [13,14,15].

## 5. Psychometric Properties of the PDMS-2

The PDMS-2 is a tool with well-established reliability and validity, confirmed across numerous studies. The authors of the original scale, Michael R. Folio and Rebecca R. Fewell, conducted the normalization study in the United States on a representative sample of 2003 children aged from birth to 71 months. The sample was diversified in terms of age, sex, ethnic background, and parental education level to ensure broad representativeness of the norms [5,6,11].

The studies were carried out across 46 U.S. locations, including daycare centers, preschools, early intervention programs, public schools, and childcare facilities. Rigorous testing procedures and standardized administration protocols were applied to minimize variability caused by environmental conditions or examiner skills. Statistical analyses confirmed high internal consistency for individual subtests (Cronbach’s alpha coefficients above 0.90), strong interrater agreement, and stable test–retest reliability [6].

Studies conducted in different populations have also demonstrated high test–retest reliability and strong interrater agreement. For example, the Persian version of the PDMS-2 showed intraclass correlation coefficients (ICC) up to 0.98 and strong correlations with the Bayley Scales (r = 0.91–0.93), confirming strong concurrent validity [16]. Similarly, a study by van Hartingsveldt et al. (2005) focusing on the fine motor subtests showed very high internal consistency (ICC 0.84–0.99) and good convergent validity with the Movement ABC (r = 0.69) [2].

According to a systematic review conducted using the COSMIN guidelines (2024), the PDMS-2 meets quality standards regarding test–retest reliability, construct validity, internal consistency, and interrater reliability. However, the review highlighted the limited number of studies examining cultural adaptations of the scale, which may affect interpretation when the tool is applied outside the U.S. population [13].

The strong psychometric properties of the PDMS-2 make it not only a widely used diagnostic tool but also a recommended measure in interventional and observational research where precise monitoring of developmental change is essential (e.g., in physical therapy, occupational therapy, and developmental psychology). The scale demonstrates high sensitivity and precision, supporting its extensive use in clinical and research settings [4,16,17]. Additionally, findings by Darrah et al. indicate that the second edition of the PDMS may be more sensitive than the original version in detecting subtle developmental delays in younger children (up to 21 months) [18] (Table 1).

Although the PDMS-2 is not formally designated as a universal gold-standard tool, it is one of the most widely used and well-validated assessments of early motor development. Its strengths include a broad age range, separate evaluation of gross and fine motor domains, detailed item scoring, and extensive evidence supporting its reliability and validity across both typical and clinical pediatric populations. For these reasons, the PDMS-2 is frequently used as a reference measure in research studies and intervention trials [13,14].

## 6. Validation and Standardization Studies of the PDMS-2 in Other Populations

In recent years, numerous studies have focused on cultural adaptation and psychometric evaluation of the PDMS-2 in populations outside the United States. Their findings provide strong evidence for the reliability and validity of the scale across diverse clinical, cultural, and linguistic contexts. The table summarizes key results from studies conducted in Brazil, Portugal, Norway, the United States, Canada, Belgium, and the Netherlands (Table 2).

Overall, these studies demonstrate that the PDMS-2 can be successfully used across various countries, provided that appropriate translation and validation procedures are implemented. High internal consistency, strong test–retest reliability, and robust construct validity support the universal applicability of the scale in clinical assessment.

## 7. PDMS-2 as an Outcome Measure in Studies on Motor Development

The PDMS-2 has been used both for clinical and research purposes. It has served as an outcome measure not only in typically developing infants but also in infants with various disorders or medical conditions affecting motor development.

### 7.1. Preterm Birth and Low Birth Weight

The PDMS-2 has been widely used to assess motor development in children born preterm, including those with very low birth weight (VLBW) and extremely low birth weight (ELBW). Studies indicate its high sensitivity in detecting subtle motor deficits that may not be visible in a standard neurological examination.

In the study by Goyen and Lui (2002), motor development was assessed in children born before 29 weeks of gestation and/or with very low birth weight (<1000 g). Although these infants did not show significant neurological abnormalities during infancy, by preschool age they achieved significantly lower scores in both gross and fine motor domains on the PDMS-2. The scale proved useful in identifying delays that were not detected during classical neurological evaluation [23].

Angelsen et al. (2001) [24] demonstrated that children with VLBW (mean birth weight 1150 g) without major neurological impairments scored lower on the PDMS-2 at 12 months corrected age, despite the absence of signs of cerebral palsy. Similarly, Sommerfelt et al. (2002) [25] confirmed significantly reduced motor function in 2-year-old VLBW children compared with controls. The most pronounced differences were observed in the “Stationary” and “Locomotion” subtests, indicating deficits primarily in gross motor skills.

In a prospective study by Evensen et al. (2009) [26], children born at 23–24 weeks of gestation obtained significantly lower PDMS-2 scores in infancy, and these deficits persisted until 5 years of age, particularly in gross motor functioning.

In the study by Cuesta-Gómez et al. (2024) [27], motor development was evaluated in 15 preterm children (mean gestational age: 32.9 weeks) and 15 full-term peers at 3–6 years of age. Preterm children achieved significantly lower scores in most PDMS-2 subscales, even though their development remained within the normative range. The scale demonstrated sensitivity in detecting subtle developmental differences between groups.

In contrast, Jeng et al. (2008) [28] assessed gait development in 29 preterm and 29 full-term children without neurological symptoms. Motor development was monitored using the PDMS-2 at 6, 12, and 18 months corrected age. Both groups achieved comparable GMQ, FMQ, and TMQ scores; however, qualitative gait analysis revealed that preterm children displayed less mature gait patterns, characterized by increased instability and poorer movement synchronization. The PDMS-2 was useful for assessing overall motor development but did not capture subtle qualitative differences observed in gait patterns.

### 7.2. Prenatal Exposure to Toxic Substances

The PDMS-2 has been used in studies examining the impact of prenatal exposure to neurotoxic substances such as cocaine and methamphetamine.

Miller Loncar et al. (2005) [29] conducted a prospective study evaluating motor development in children prenatally exposed to cocaine (*n* = 392) and a control group (*n* = 776) from 1 to 18 months of age. At 18 months, exposed children achieved significantly lower scores in both gross and fine motor domains on the PDMS-2. The scale proved useful in detecting subtle functional deficits.

As part of the IDEAL study in New Zealand, Wouldes et al. (2014) [30] compared children prenatally exposed to methamphetamine (MA) (*n* = 103) with a control group (*n* = 107). After adjusting for confounding variables, exposed children scored on average 3.2 points lower in gross motor development on the PDMS-2 than their peers. Additionally, male sex and Māori ethnicity were predictors of lower motor scores.

In another component of the IDEAL project, Smith et al. (2011) [31] evaluated motor development in MA-exposed children (*n* = 204) and controls (*n* = 208) at ages 1 and 3 years. Exposed children scored significantly lower in the “Grasping” subtest as early as the first year of life (*p* = 0.018), and these deficits persisted to age 3. In contrast, psychomotor indices measured with the BSID-2 did not show significant differences between groups. This illustrates the high sensitivity of the PDMS-2 in detecting subtle motor effects of prenatal neurotoxic exposure.

### 7.3. Genetic Syndromes and Metabolic Disorders

The PDMS-2 has also been applied in research involving children with genetic syndromes and metabolic disorders. The scale has proven valuable for monitoring therapeutic progress and identifying motor deficits in these clinical populations.

In the study by Connolly et al. (2006), 17 children with Down syndrome (DS), aged 4 to 36 months, were evaluated using the PDMS-2 and compared with the BSID-2. The PDMS-2 demonstrated strong concurrent validity with the BSID-II for both gross motor (r = 0.85) and fine motor skills (r = 0.79) [4]. Children with DS obtained lower raw and standardized scores compared with the normative sample, with the largest deficits observed in locomotion and object manipulation [4].

Additional support for the usefulness of the PDMS-2 in children with DS can be found in an abstract published in Pediatric Physical Therapy (2004) [32], which compared gross motor subtest scores in this population. Although detailed numerical data were not provided, it remains one of the few early references addressing the use of PDMS-2 in children with Down syndrome.

In Dusing et al. (2007) [33], the motor development of four children with Hurler syndrome (MPS I), aged 6 to 24 months, was assessed during enzyme replacement therapy and bone marrow transplantation. The PDMS-2 served as the primary measurement tool to track changes in gross and fine motor skills throughout individualized therapy programs. The scale captured progression over time and revealed substantial individual differences, but all children showed increases in raw scores across repeated assessments, supporting the PDMS-2 as a sensitive tool for monitoring outcomes in rare genetic disorders.

Wu et al. (2011) [34] evaluated 41 children with phenylketonuria (PKU), aged 12–35 months, treated with dietary management from the neonatal period. Their PDMS-2 results did not differ significantly from the control group. The authors concluded that children with adequately treated PKU exhibit typical motor development, although greater variability was observed in temperament and emotional functioning.

In a study by Hwu et al. (2025) [35], gene therapy for aromatic L-amino acid decarboxylase deficiency (AADCd) was evaluated using the PDMS-2 total score as an outcome measure. After 18 months of treatment, 86% of children achieved an improvement of at least 40 points—considered clinically meaningful. The scale demonstrated high sensitivity to change and strong correlations with cognitive and neuromotor outcomes (Bayley-III, GMFM), confirming its value in this rare condition.

Klingels et al. (2015) [36] assessed the usefulness of the PDMS-2 in monitoring motor development in young boys with Duchenne muscular dystrophy (DMD). Despite motor scores falling below age norms, the PDMS-2 enabled identification of early deficits and documentation of disease progression. The authors concluded that the scale is suitable for screening and interventional research in this population.

Hoskens et al. (2024) [37] evaluated 17 boys with DMD aged 11 months to 6 years. The PDMS-2 was used to quantify gross motor skills and to classify motor functioning relative to age norms. Significant deficits were detected, with more than 40% of participants scoring below the 5th percentile. The authors emphasized the scale’s usefulness for early detection of motor impairments in DMD.

Another study by Connolly et al. (2012) [20] assessed children with various developmental disorders (including genetic conditions) aged 12–26 months. Strong correlations between PDMS-2 and Bayley-3 scores were reported, confirming the validity of the PDMS-2 for evaluating motor function in children with heterogeneous neurodevelopmental conditions.

Finally, the systematic review by Zhu et al. (2024), conducted in accordance with COSMIN guidelines, found that the PDMS-2 was used across multiple genetic syndromes (e.g., Down, Prader–Willi, Williams), with the tool demonstrating high content validity, structural validity, and reliability (ICC > 0.90) in these populations [13].

### 7.4. Cerebral Palsy and Neurodevelopmental Disorders

The PDMS-2 has been widely used as an adjunct tool in research involving children with cerebral palsy (CP) and other neurodevelopmental disorders, particularly for evaluating intervention effectiveness and profiling motor function.

In the study by Wang et al. (2006) [22], the psychometric properties of the PDMS-2 were examined in relation to children with CP. The study included 45 children with bilateral CP aged 1 to 72 months. The aim was to evaluate internal consistency, sensitivity to change, and responsiveness of the tool. The results confirmed high reliability (ICC > 0.90) and strong responsiveness—the scale was capable of detecting changes in motor performance over the course of physical therapy intervention. This remains one of the most thoroughly documented studies validating the use of the PDMS-2 in children with CP.

Darrah et al. (2007) [19] evaluated the effectiveness of an intervention program aimed at promoting spontaneous lower-limb activity in infants at risk for CP. Forty infants (mean age: 4.6 months) were enrolled, with 20 assigned to the intervention group and 20 to a control group receiving standard care. The PDMS-2 was administered every two months over a 12-month period. Children in the intervention group demonstrated a faster rate of improvement in the “Gross Motor” subscale, suggesting potential benefits of early targeted lower-limb interventions.

In a systematic review by Burgess et al. (2019) [38], the PDMS-2 was identified as one of the few observational tools with evidence supporting its reliability and validity for evaluating upper limb activity in children with bilateral CP. Although the PDMS-2 is not dedicated specifically to upper limb assessment, its “Grasping” and “Visual-Motor Integration” subtests can provide useful information about manual dexterity and functional hand use.

A single case study by George and Elchert (2007) [39] explored the effects of orthotic intervention in a 20-month-old child with hydrocephalus and developmental delay. The PDMS-2 was used to evaluate outcomes following 12 weeks of ankle-foot orthosis use. Improvements were observed in the “Gross Motor” and “Locomotion” subtests, demonstrating the tool’s utility in documenting therapeutic progress in nonstandard clinical cases.

Provost et al. (2007) [3] examined motor development in children aged 21–41 months across three groups: autism spectrum disorder (ASD), developmental delay (DD), and developmental concerns (DC). The PDMS-2 identified significant differences among the groups, with children with ASD achieving the lowest scores on fine motor and visual-motor integration tasks. The authors recommended the PDMS-2 as a sensitive tool for detecting subtle differences in motor functioning in children with neurodevelopmental disorders.

### 7.5. Human Immunodeficiency Virus (HIV)

Chronic HIV infection can adversely affect child development both through direct effects on the central nervous system and indirectly through long-term treatment burden, malnutrition, or psychosocial factors. The PDMS-2 has been used in studies evaluating motor development in children infected with HIV.

Smith et al. (2002) [40] assessed motor development in 143 children with HIV up to 5 years of age using the PDMS-2, with a subgroup of 22 children followed longitudinally over 18 months. Children scored significantly below age norms across most gross and fine motor subtests, particularly in visual-motor integration and balance. The “Grasping” and “Object Manipulation” subtests were exceptions, showing scores closer to normative values. The PDMS-2 enabled detailed characterization of the motor profile of children with HIV and highlighted the need for targeted intervention and support.

Jelsma et al. (2011) [41] evaluated motor development in 44 orphaned children aged 3–6 years, of whom 23 were HIV-positive. The PDMS-2 was administered twice (baseline and after 6 months). HIV-positive children scored significantly lower on GMQ, FMQ, and TMQ compared with HIV-negative peers. The PDMS-2 effectively detected group differences related to health status and caregiving context (institutional vs. family-based care).

### 7.6. Oncological Conditions

Children undergoing cancer treatment are at risk of developing secondary motor impairments due to neurotoxic effects of chemotherapy, prolonged immobilization, malnutrition, or the impact of the malignancy itself. In this group, the PDMS-2 has been used to quantify motor deficits and monitor the effects of physical therapy and medical interventions.

Mitchell et al. (2005) [42] evaluated motor development in 47 children with opsoclonus–ataxia syndrome (OAS) associated with neuroblastoma. The PDMS-2 was used repeatedly to measure both gross and fine motor function. The scale enabled tracking of motor improvements following immunosuppressive treatment. The authors highlighted its responsiveness and applicability in monitoring neurological recovery in pediatric autoimmune and paraneoplastic conditions.

### 7.7. Congenital Heart Defects (CHD)

Children with congenital heart defects (CHD) represent a high-risk group for motor delays resulting from underlying cardiac pathology and its treatment—including surgical intervention, prolonged hospitalization, or chronic cardiorespiratory insufficiency. The PDMS-2 has been used to assess motor outcomes and intervention efficacy in this population.

Du et al. (2015) [43] evaluated motor development in infants with CHD following catheterization procedures. A total of 147 newborns younger than one month corrected age were included. The goal was to compare the effectiveness of passive and active exercise programs—implemented by therapists or parents—against natural development in a non-intervention control group. The PDMS-2 was administered at five time points: 1, 4, 6, 12, and 24 months of age. All composite scores (GMQ, FMQ, TMQ) were analyzed. The authors concluded that the PDMS-2 is a valid and meaningful tool for monitoring motor development and intervention outcomes in infants with cardiac conditions.

### 7.8. Environmental Factors

Motor development is shaped not only by biological factors but also by environmental influences such as socioeconomic status, family lifestyle, caregiving practices, and access to developmental stimulation. Two studies examined these associations using the PDMS-2.

Majnemer and Barr (2006) [44] assessed the effects of sleep position on motor development in healthy infants at 4 and 6 months of age. Infants placed in the supine position for sleep (per SIDS prevention guidelines) achieved significantly lower PDMS-2 scores—especially in gross motor skills—compared with infants sleeping prone. Some infants demonstrated developmental delay. The authors emphasized the importance of considering sleep position when interpreting motor assessments and recommended increased daytime prone positioning (“tummy time”) for infants sleeping supine. The study highlighted the sensitivity of the PDMS-2 to subtle environmental influences.

Piñero-Pinto et al. (2020) [45] evaluated the relationship between motor development and visual functions in 38 typically developing children aged 12 to 36 months. Based on PDMS-2 results, children were categorized into fast and slow motor development groups. Children with slower motor development exhibited significantly weaker visual functions (e.g., visual-motor integration, saccadic movements). The PDMS-2 effectively differentiated motor development levels within a typically developing population.

## 8. Limitations of the PDMS-2

Although the PDMS-2 is widely used and supported by substantial psychometric evidence, several limitations should be considered when selecting this tool for clinical or research purposes. First, the instrument is standardized only for children aged 0–71 months. As a result, its applicability is restricted in older preschoolers and children of school age who continue to present motor concerns, and alternative tools such as the MABC-2 or TGMD-2/3 may be more appropriate in these cases [6,46,47]. Second, because the PDMS-2 uses a performance-based scoring approach, it may be less sensitive to subtle differences in motor control, postural stability, or movement variability. Observational tools designed specifically to evaluate postural alignment and movement patterns in infancy, such as the AIMS, may detect early deviations more readily. These considerations have been highlighted in recent systematic reviews comparing the sensitivity and discriminative properties of motor assessment tools [13,15,48].

Third, the PDMS-2 may be less suitable for children with very severe motor impairments, particularly those who are unable to attempt many of the required items, which can lead to floor effects and reduced interpretability of results. Floor effects for PDMS-2 subtests have been reported in cohorts with rare, severe neurogenetic disorders, where most children cluster at the lowest end of the scoring range despite clinically observable variability in motor function [49,50,51]. Furthermore, systematic reviews have highlighted that the discriminative and predictive properties of the PDMS-2 may be reduced in certain clinical groups, such as children with mild developmental concerns or those undergoing long-term follow-up after neonatal complications [13,14,15]. Finally, cultural and population differences in motor trajectories underline the need for continuous standardization and validation of the PDMS-2 across diverse regions, as normative discrepancies may influence score interpretation [5,11].

Taken together, these limitations underscore the importance of selecting assessment instruments that match both the developmental profile of the child and the clinical purpose of the evaluation. The PDMS-2 remains a valuable tool, but its use should be complemented with other measures when detailed qualitative analysis, assessment beyond the preschool period, or evaluation of severe motor impairment is required.

## 9. Conclusions

The PDMS-2 is a standardized tool for assessing motor development in children from birth to 5 years of age. It is widely used in both clinical practice and research. The scale has been applied as an outcome measure in randomized trials, interventional studies, and observational research involving preterm infants, children with genetic syndromes, metabolic disorders, congenital heart defects, HIV infection, and children exposed to adverse environmental conditions.

The PDMS-2 has been standardized and/or validated in numerous countries across multiple continents; however, further research is needed to confirm its full psychometric robustness in Central and Eastern European populations. Key strengths of the PDMS-2 include its wide age range, ability to assess both gross and fine motor skills, and its precise, quantitative scoring system.

Overall, the PDMS-2 remains a useful and versatile tool for evaluating motor development across diverse clinical and research contexts.

The recent release of the Peabody Developmental Motor Scales—Third Edition (PDMS-3) in 2023 introduces updated normative data and a revised subtest structure. However, empirical studies applying the PDMS-3 in clinical or longitudinal contexts are still limited. As a result, the current body of evidence on psychometric properties, diagnostic accuracy, and clinical utility remains largely based on the PDMS-2. Future research directly comparing the second and third editions will be essential to determine the impact of these updates on motor assessment and to clarify whether the PDMS-3 should replace the PDMS-2 in routine clinical and research settings [12].

## Figures and Tables

**Table 1:** Studies Assessing the Concurrent Validity and Reliability of the PDMS-2 in North American Populations (USA and Canada).

Authors (Year)	Population	Study Type	N	Number of Raters	Interrater Reliability	Referential Tool	Concurrent Validity
Connolly et al. (2012) [19]	USA—children with developmental delay, 29 days to 2 years	Concurrent validity: PDMS-2 vs. Bayley-3	48	not reported	not reported	BSID-3	r = 0.69–0.95
Darrah et al. (2007) [18]	Canada—children aged ≥ 9 months	Reliability and validity	77	13		PDMS 1 edition	r = 0.71–0.75
Connolly et al. (2006) [4]	USA—12-month-old infants	Tool validity	15	6	not reported	BSID-2	r = 0.84–0.87

BSID-2—Bayley Scales of Infant Development, Second Edition; BSID-3—Bayley Scales of Infant and Toddler Development, Third Edition.

**Table 2:** Psychometric properties of culturally adapted versions of the PDMS-2.

Authors (Year)	Country/Population	Translation	Study Type	N	Number of Raters	Interrater Reliability	Test–Retest Reliability	Referential Tool	Concurrent Validity
Zhu et al. (2024) [13]	review according to COSMIN	various language versions	systematic review	22 articles	not applicable	assessment using COSMIN criteria	—	various tools	varied, but rated as sufficient
Ustad et al. (2023) [17]	Norway—preterm infants aged 6 and 24 months	Norwegian	concurrent and predictive validity	139	2	ICC = 0.89–0.93	—	AIMS, BSID-3	r = 0.83–0.91 (AIMS), r = 0.70–0.79 (BSID-3)
Zanella et al. (2021) [11]	Brazil—children aged 1–71 months	Portuguese	cultural adaptation and psychometric analysis	637	3	ICC = 0.97–0.99	—	none	—
Rebelo et al. (2020) [5]	Portugal—children aged 12–48 months	Portuguese	cultural adaptation and psychometric analysis	392	2	ICC = 0.98–0.99, α = 0.84–0.97	—	none	—
Abdelazeim et al. (2016) [20]	Egypt—healthy children aged 36–42 months	original version	assessment of cultural validity for fine motor subtests	195	not reported	not reported	—	comparison with normative values for the original population (USA, Canada)	—
Tavasoli et al. (2014) [16]	Iran—preterm and full-term children aged 17–20 months	Persian	reliability and validity	88	2	ICC = 0.98	—	BSID-2 (PDI)	r = 0.91 (FM) and r = 0.93 (GM); analysis of groups differing in birth weight
Van Waelvelde et al. (2007) [21]	Belgium—children aged 4–5 years	Dutch	convergent validity	31	not reported	not reported	—	M-ABC	r = 0.69 (PDMS-2 total vs. M-ABC manual dexterity)
Wang & Liao (2006) [22]	Taiwan—children with cerebral palsy (CP), aged 1–6 years	Chinese (traditional)	sensitivity and responsiveness of the PDMS-2 after intervention	25	2	ICC = 0.95–0.99	—	none	not applicable (responsiveness study)
Van Hartingsveldt et al. (2005) [2]	Netherlands—children aged 4–5 years with and without mild fine motor difficulties	Dutch	reliability (test–retest, interrater, convergent validity)	36	2	r = 0.94–0.99 (interrater), r = 0.84–0.98 (test–retest)	—	M-ABC	r = 0.69 (manual dexterity—convergent validity)

AIMS—Alberta Infant Motor Scale; BSID-2—Bayley Scales of Infant Development, Second Edition; BSID-3—Bayley Scales of Infant and Toddler Development, Third Edition; FM—Fine Motor; GM—Gross Motor; M-ABC—Movement Assessment Battery for Children; ICC—Intraclass Correlation Coefficient; PDI—Psychomotor Development Index; COSMIN—Consensus-Based Standards for the Selection of Health Measurement Instruments.

## Data Availability

No new data were created or analyzed in this study. Data sharing is not applicable to this article.
